# Characterization of a GHF45 cellulase, AkEG21, from the common sea hare *Aplysia kurodai*

**DOI:** 10.3389/fchem.2014.00060

**Published:** 2014-08-06

**Authors:** Mohammad M. Rahman, Akira Inoue, Takao Ojima

**Affiliations:** ^1^Laboratory of Marine Biotechnology and Microbiology, Division of Applied Marine Life Science, Graduate School of Fisheries Sciences, Hokkaido UniversityHakodate, Japan; ^2^Department of Fisheries Biology and Genetics, Bangladesh Agricultural UniversityMymensingh, Bangladesh

**Keywords:** *Aplysia kurodai*, AkEG21, endo-β-1,4-glucanase, cellulase, GHF45, cDNA cloning, primary structure, phylogenic analysis

## Abstract

The common sea hare *Aplysia kurodai* is known to be a good source for the enzymes degrading seaweed polysaccharides. Recently four cellulases, i.e., 95, 66, 45, and 21 kDa enzymes, were isolated from *A. kurodai* (Tsuji et al., 2013). The former three cellulases were regarded as glycosyl-hydrolase-family 9 (GHF9) enzymes, while the 21 kDa cellulase was suggested to be a GHF45 enzyme. The 21 kDa cellulase was significantly heat stable, and appeared to be advantageous in performing heterogeneous expression and protein-engineering study. In the present study, we determined some enzymatic properties of the 21 kDa cellulase and cloned its cDNA to provide the basis for the protein engineering study of this cellulase. The purified 21 kDa enzyme, termed AkEG21 in the present study, hydrolyzed carboxymethyl cellulose with an optimal pH and temperature at 4.5 and 40°C, respectively. AkEG21 was considerably heat-stable, i.e., it was not inactivated by the incubation at 55°C for 30 min. AkEG21 degraded phosphoric-acid-swollen cellulose producing cellotriose and cellobiose as major end products but hardly degraded oligosaccharides smaller than tetrasaccharide. This indicated that AkEG21 is an endolytic β-1,4-glucanase (EC 3.2.1.4). A cDNA of 1013 bp encoding AkEG21 was amplified by PCR and the amino-acid sequence of 197 residues was deduced. The sequence comprised the initiation Met, the putative signal peptide of 16 residues for secretion and the catalytic domain of 180 residues, which lined from the N-terminus in this order. The sequence of the catalytic domain showed 47–62% amino-acid identities to those of GHF45 cellulases reported in other mollusks. Both the catalytic residues and the N-glycosylation residues known in other GHF45 cellulases were conserved in AkEG21. Phylogenetic analysis for the amino-acid sequences suggested the close relation between AkEG21 and fungal GHF45 cellulases.

## Introduction

Cellulose, a structural polysaccharide comprising 1,4-linked β-D-glucopyranose residues, exists mainly in plant cell wall as crystalline microfibrils (Jagtap and Rao, 2005). Since plant cellulose accounts for almost a half of total carbohydrate biomass on the Earth, intensive uses of the cellulose are expected to solve various problems that we are facing in ecological, environmental and energy fields (Agbor et al., 2011; Yang et al., 2011). In this respect, degradation of cellulosic materials by cellulose-degrading enzymes will be a fundamentally important technique because the cellulose-degrading enzyme can convert insoluble cellulose to soluble oligosaccharides and glucose without consuming high energy and producing hazardous byproducts (Michel and Czjzek, 2013; Ojima, 2013; Tsuji et al., 2013). The resulted sugars are applicable for foods, feeds, pharmaceutics, fermentation substrates, etc.

Complete enzymatic degradation of cellulose is usually achieved by the collaborative actions of three enzymes, namely, (1) endo-β-1,4-glucanase (EC 3.2.1.4) which randomly cleaves internal β-1,4-linkages of amorphous regions in cellulose fibers, (2) cellobiohydrolase (EC 3.2.1.91) which releases cellobiosyl unit from non-reducing end of cellulose chain, and (3) β-D-glucosidase (EC 3.2.1.21) which releases glucose unit from cello-oligosaccharides (Lynd et al., 2002; Perez et al., 2002; Bayer et al., 2004). Although individual enzyme alone cannot completely depolymerize crystalline cellulose, the synergistic action of three enzymes efficiently promotes the depolymerization of cellulose. Among the three enzymes, endo-β-1,4-glucanase is the primarily important for the depolymerization of cellulose since it first acts on cellulose and provides new substrate sites for cellobiohydrolase and β-D-glucosidase. Accordingly, endo-β-1,4-glucanase is generally called “cellulase.” Fungal and microbial cellulases have already been used in various purposes, e.g., detergent, textile, food, paper, pulp, brewing and winery (Sheehan and Himmel, 1999; Bhat, 2000; Zaldivar et al., 2001; Kuhad et al., 2011; Mojsov, 2012). Cellulases are also expected as a biocatalyst in the production of biofuels from cellulose. If fermentable sugars can be produced from unused cellulosic materials at low cost, food-fuel conflicts in the bioethanol production using edible crops will be circumvented.

Cellulase distributes over various organisms, e.g., archaea (Gueguen et al., 1997; Li et al., 2003), bacteria (Tomme et al., 1995; Hong et al., 2002; Masuda et al., 2006; Fibriansah et al., 2007), fungi (de la Cruz et al., 1995; Tomme et al., 1995), plants (Pesis et al., 1978; Castresana et al., 1990), and herbivorous invertebrates such as termite, cockroach, crayfish and mollusks (Watanabe et al., 1998; Yan et al., 1998; Byrne et al., 1999; Tokuda et al., 1999; Watanabe and Tokuda, 2001; Xu et al., 2001; Sugimura et al., 2003; Suzuki et al., 2003; Davison and Blaxter, 2005; Nishida et al., 2007; Sakamoto et al., 2007; Sakamoto and Toyohara, 2009; Tsuji et al., 2013). Previously, cellulase activities detected in the invertebrate animals were considered to be originated from symbiotic microbes in their digestive tracts or contamination by foods (Cleveland, 1924; Martin and Martin, 1978). However, recent biochemical and genomic studies have revealed that cellulases found in insects, crustaceans, annelids, mollusks, echinoderms and nematodes are their own gene products.

To date, a large number of primary structures of cellulases have been enrolled in CAZy data base (Cantarel et al., 2009). These cellulases have been classified under GHF (glycosyl hydrolase family) 5, 6, 7, 8, 9, 10, 11, 12, 26, 44, 45, 48, 51, and 74 on the basis of hydrophobic cluster analysis for amino-acid sequences (Henrissat et al., 1989; Henrissat, 1991; Henrissat and Bairoch, 1993). Invertebrate cellulases are enrolled in five families, i.e., GHF5 (nematodes: *Globodera rostochiensis* and *Heterodera glycines*; Smant et al., 1998), GHF6 (sea squirt: *Ciona savignyi*; Matthysse et al., 2004), GHF9 (termite: *Reticulitermes speratus*, Watanabe et al., 1998; abalone: *Haliotis discus hannai*, Suzuki et al., 2003; sea urchin: *Strongylocentrotus nudus*, Nishida et al., 2007), GHF10 (freshwater snails: *Ampullaria crossean*, Wang et al., 2003; *Pomacea canaliculata*, Imjongjirak et al., 2008), and GHF45 (bivalve: *Mytilus edulis*, Xu et al., 2001; freshwater snail: *A. crossean*, Guo et al., 2008; freshwater bivalve: *Corbicula japonica*, Sakamoto and Toyohara, 2009). Among these cellulases, GHF9-type cellulases appear to be most widespread in nature and well characterized (Davison and Blaxter, 2005). In molluscan cellulases, GHF9 enzyme was identified in *H. discus hannai* (Suzuki et al., 2003) and *A. kurodai* (Tsuji et al., 2013), while both GHF10 and GHF45 enzymes were identified in *P. canaliculata* (Imjongjirak et al., 2008) and *A. crossean* (Ding et al., 2008), and GHF45 enzymes were identified in *M. edulis* (Xu et al., 2001), *A. crossean* (Guo et al., 2008), *C. japonica* (Sakamoto and Toyohara, 2009) and *A. kurodai* (Tsuji et al., 2013). Some mollusks possess plural cellulases, e.g., GHF9 and GHF45 cellulases (Sakamoto et al., 2007; Guo et al., 2008; Li et al., 2009; Sakamoto and Toyohara, 2009; Tsuji et al., 2013). The synergistic action of multiple enzymes appeared to improve the production of glucose from seaweed cellulose (Tsuji et al., 2013). Among the molluscan cellulases, GHF45 enzyme has been characterized by its smaller molecular size compared with other cellulases. Namely, the molecular size of GHF45 enzymes is ~25 kDa, while those of GHF9 and GHF10 enzymes are 45–63 kDa. The small size of GHF45 cellulases appeared to be advantageous in performing protein-engineering and crystallography studies, since low molecular mass proteins are usually heat stable and easily produced by heterogeneous expression systems. Actually, the GHF45-type cellulase CjCel45 from freshwater clam was successfully produced by the *Escherichia coli* expression system (Sakamoto and Toyohara, 2009) and the three-dimensional structure of Cel45A from *M. edulis* could be analyzed by X-ray crystallography (PDB ID, 1WC21006).

To date, GHF45 cellulase genes have been identified only in a few mollusks (Xu et al., 2001; Guo et al., 2008; Sakamoto and Toyohara, 2009) and enzyme proteins have been isolated only from *M. edulis* (Xu et al., 2000), *A. crossean* (Li et al., 2005), and *A. kurodai* (Tsuji et al., 2013). Molluscan GHF45 cellulases were suggested to be acquired by horizontal gene transfer from fungi by phylogenetic analyses (Scholl et al., 2003; Kikuchi et al., 2004; Sakamoto and Toyohara, 2009); however, accumulation of primary structure data seems to be still insufficient for detailed discussion about the origin and molecular evolution of molluscan GHF45 cellulases.

The common sea hare *A. kurodai* is a good source for polysaccharide-degrading enzymes since it harbor much digestive fluid in gastric lumen (Kumagai and Ojima, 2009; Rahman et al., 2010; Zahura et al., 2010; Tsuji et al., 2013; Kumagai et al., 2014). Recently, four cellulase isozymes, i.e., 21, 45, 66, and 95 K cellulases, were isolated from the digestive fluid of *A. kurodai* (Tsuji et al., 2013). Among these enzymes, the 21K enzyme was suggested to be GHF45 cellulase. We also had noticed that the digestive fluid of *A. kurodai* contained plural cellulases and the smallest enzyme was of ~21 kDa. This enzyme was considered to correspond to the 21K cellulase reported by Tsuji et al. (2013). Although partial amino-acid sequences of the 21K cellulase were reported, no entire primary structure has been determined yet.

In the present study, we isolated the ~21 kDa enzyme from the digestive fluid of *A. kurodai* and investigated its general properties. Further, we cloned the cDNA encoding this enzyme and confirmed that this enzyme is a member of GHF45. This cDNA will provide the basis for protein-engineering studies on *Aplysia* GHF45 cellulase.

## Materials and methods

### Materials

Sea hares identified as *A. kurodai* (average body length and weight; ~15 cm and ~400 g, respectively) were collected in the shore of Hakodate, Hokkaido Prefecture of Japan in July 2012. Approximately 112 mL of digestive fluid was obtained from the gastric lumen of 14 animals after dissection. The digestive fluid was dialyzed against 2 mM sodium phosphate buffer (pH 7.0) for 2 h and centrifuged at 10,000 × g for 10 min to remove insoluble materials. The supernatant (crude enzyme) was used for purification of cellulase. Carboxymethyl cellulose (CMC, medium viscosity) was purchased from ICN Bio medicals, Inc. (OH, USA). TOYOPEARL CM-650M was purchased from Toyo Soda Mfg, Co. (Tokyo, Japan) and Superdex 200 10/300 GL from GE Healthcare UK Ltd. (Little Chalfont, Buckingham shire, England). Cellooligosaccharides (disaccharide – hexasaccharide, G2 – G6) were prepared by limited acid hydrolysis. Briefly, 1 g of cellulose powder (Wako Pure Chemical Industries Co. Ltd. Osaka, Japan) was hydrolyzed with 100 mL of 0.2 N HCl at 100°C for 1 h, and the supernatant containing cellulose fragments was neutralized with 1 N NaOH. Approximately 50 mg of cellulose fragments were subjected to gel-filtration through a column of BioGel-P2 (2 × 100 cm) and cellooligosaccharides were separately eluted with 10 mM sodium phosphate buffer (pH 7.0) and stored at −20°C until use. RNAiso Plus and Oligotex dT30 were purchased from TaKaRa (Tokyo, Japan). cDNA synthesis kit and 5′- and 3′-Full RACE kits were purchased from TaKaRa and TA-PCR cloning kit comprising pTAC-1 and *E. coli* DH5α was from Biodynamics (Tokyo, Japan). Restriction endonucleases, T4 DNA ligase, agarose, *E. coli* strain DH5α were purchased from TaKaRa. AmpliTaq Gold PCR Master Mix and BigDye-Terminator Cycle Sequencing kit were from Applied Biosystems (Foster city, CA, USA). Bacto-tryptone, Bacto-yeast extract and other reagents were from Wako Pure Chemicals Industries Ltd. (Osaka, Japan).

### Purification of *A. kurodai* cellulase

Crude enzyme from *A. kurodai* (~100 mL) was first subjected to ammonium sulfate fractionation. Cellulase activity was detected in the fraction precipitated between 40 and 60% saturation of ammonium sulfate. This fraction was collected by centrifugation at 10,000 × g for 20 min, dissolved in and dialyzed against 10 mM sodium phosphate buffer (pH 7.0) for 24 h. The dialysis bag was changed every 2 h to avoid puncturing by cellulase action. The dialysate was then applied to a TOYOPEARL CM-650M column (1.5 × 20 cm) pre-equilibrated with the same buffer. Proteins adsorbed to the column were developed by linear gradient of NaCl from 0 to 0.3 M. Fractions showing cellulase activity were pooled and dialyzed against 10 mM sodium phosphate buffer (pH 7.0) and lyophilized. The dried material was dissolved in 0.05 M NaCl—10 mM sodium phosphate buffer (pH 6.0) and subjected to AKTA-FPLC (GE Healthcare) equipped by Superdex 200 10/300 GL column. Cellulase was eluted with 0.05 M NaCl—10 mM sodium phosphate buffer (pH 6.0) at a flow rate of 1 mL/min.

### Assay for cellulase activity

Standard assay for cellulase activity was carried out with a reaction mixture containing 0.5% CMC, 10 mM sodium phosphate (pH 6.0), and 0.01–0.1 mg/mL of enzyme at 30°C. Reducing sugar released by the reaction was determined by the method of Park and Johnson (1949). One unit of cellulase activity was defined as the amount of enzyme that produces reducing sugar equivalent to 1 μmol of glucose per 1 min. Temperature dependence of the cellulase was determined at 10–70°C and pH 6.0. pH dependence was determined at 30°C in reaction mixtures adjusted to pH 4.0–10.0 with 50 mM sodium phosphate. Thermal stability was assessed by measuring the residual activity in the standard assay condition after heating at 10–70°C for 30 min. The average values of triplicate measurements were shown with standard deviations.

### Thin-layer chromatography

Thin-layer chromatography (TLC) for degradation products of cellulose and cellooligosaccharides was carried out with Silica gel-60 TLC plates (Merck KGaA, Darmstadt, Germany). Two μL of the degradation products (~5 mg/mL) were applied to the TLC plate and developed with 1-butanol/acetic acid/water (2:1:1, v/v/v). The sugars separated on the plate were detected by heating at 120°C for 15 min after spraying 10% (v/v) sulfuric acid in ethanol.

### SDS-PAGE

SDS-PAGE was carried out by the method of Porzio and Pearson (1977) using 10% (w/v) polyacrylamide gel containing 0.1% (w/v) SDS. After the electrophoresis, the gel was stained with 0.1% (w/v) Coomassie Brilliant Blue R-250–50% (v/v) methanol–10% (v/v) acetic acid, and the background of the gel was destained with 5% (v/v) methanol–7% (v/v) acetic acid. Molecular masses of proteins were estimated with a Protein Marker, Broad Range (New England Biolabs, Inc. MA, USA).

### Protein concentration

Protein concentration was determined by either the biuret method (Gornall et al., 1949) or the method of Lowry et al. (1951) using bovine serum albumin fraction V as a standard protein.

### Determination of partial amino-acid sequences

The N-terminal amino-acid sequence of cellulase was determined with specimens electro-blotted to polyvinylidene difluoride membrane and ABI 492 protein sequencer (Applied Biosystems). The internal amino-acid sequences of cellulase were determined by mass spectrometry with tryptic and lysylendopeptidyl fragments prepared by the digestion with 1/200 (w/w) enzymes at 37°C for 12 h. The fragments were subjected to matrix-assisted laser desorption ionization-time of flight mass spectrometry (MALDI-TOF MS) using Proteomics Analyzer 4700 (Applied Biosystems) and the amino-acid sequences of the fragments were analyzed by MS/MS mode with DeNovo Explorer software (Applied Biosystems). Homology searches for the amino-acid sequences to the public databases were performed with the BLAST program (http://blast.ddbj.nig.ac.jp/top-j.html) provided by DNA Data Bank of Japan.

### cDNA cloning

Total RNA was extracted from ~0.1 g of hepatopancreas of *A. kurodai* using RNAiso Plus and mRNA was selected from the total RNA with Oligotex-dt(30) (TaKaRa). Hepatopancreas cDNA was synthesized from the mRNA with a cDNA synthesis kit (TaKaRa) using random hexanucleotide primers. Cellulase cDNAs were amplified by the PCR using the hepatopancreas cDNA and degenerated primers synthesized on the basis of partial amino-acid sequences. PCR was performed in 20 μL of reaction mixture containing 50 mM KCl, 15 mM Tris–HCl (pH 8.0), 0.2 mM each of dATP, dTTP, dGTP, and dCTP, 2.5 mM MgCl_2_, 5 pmol/μL primers, 1 ng/μL template DNA, and 0.5 units AmpliTaq Gold DNA polymerase (Applied Biosystems). A successive reaction consisting of 94°C for 30 s, 55°C for 30 s, and 72°C for 60 s was repeated 40 cycles with Thermal Cycler Dice mini (TaKaRa). Sizes of amplified cDNAs were estimated by 1.2% agarose-gel electrophoresis and the target cDNAs were excised from the gel and cloned with TA-PCR cloning kit (Invitrogen). The transformed *E. coli* was grown in 2 × YT medium supplemented by 50 μg/mL ampicillin at 37°C for 14 h with shaking at 150 rpm/min. The plasmids extracted from the transformants were subjected to sequence analysis with BigDye-Terminator Cycle Sequencing kit and ABI 3130*xl* Genetic Analyzer (Applied Biosystems). The 3′-RACE and 5′-RACE PCRs were carried out with specific primers synthesized on the basis of nucleotide sequences of above amplified cDNAs with a successive reaction at 94°C for 30 s, 57°C for 30 s, and 72°C for 1.0 min, which was repeated 30 cycles. The amplified DNAs were cloned and sequenced as described above.

### Phylogenetic analysis for GHF45 cellulases

Phylogenetic analysis was carried out with amino-acid sequence data of *A. kurodai* cellulase and other GHF45 cellulases which are enrolled in GenBank (http://www.ncbi.nlm.nih.gov/) and CAZy database (http://www.cazy.org/fam/acc_GH.html). The amino-acid sequences of GHF45 cellulases were first aligned with ClustalW2 program (Larkin et al., 2007). The alignment was then corrected by trimming the sequences with Gblocks (Castresana, 2000; Talavera and Castresana, 2007). The maximum-likelihood algorithm implemented in MEGA6 software (Tamura et al., 2013) was used to generate phylogenetic tree. The bootstrap values were calculated from 1,000 replicates.

## Results

### Isolation and characterization of *Aplysia* 21 kDa cellulase

**Figure 1:**
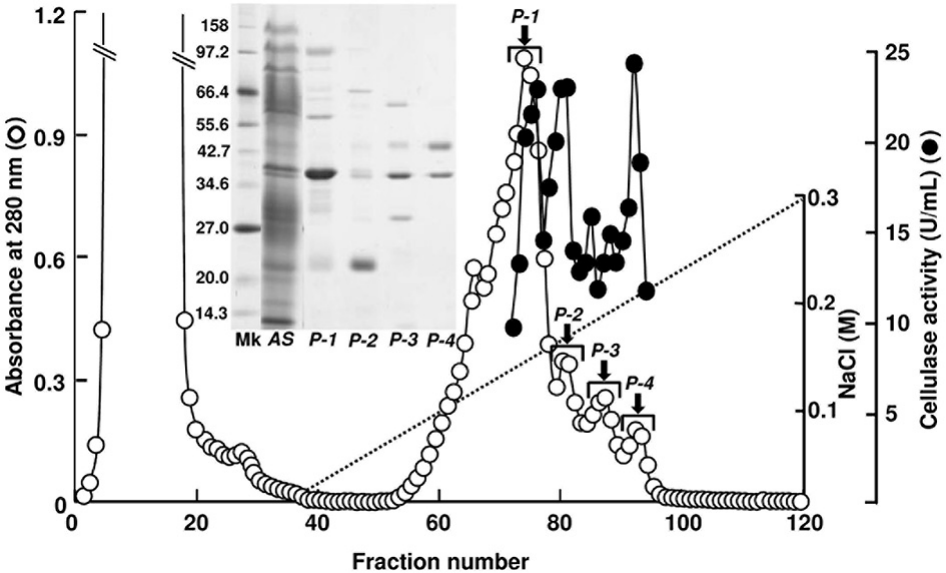
**TOYOPEARL CM-650M column chromatography for *A. kurodai* cellulase**. Proteins precipitated between 40 and 60% saturation of ammonium sulfate from the crude enzyme was applied to a TOYOPEARL CM-650M column (1.5 × 20 cm) and eluted with a linear gradient of 0–0.3 M NaCl in 10 mM sodium phosphate buffer (pH 7.0) at a flow rate of 15 mL/h. Each fraction contains 7.0 mL. SDS-PAGE of the peak fractions are shown in the inset.

Cellulase activity was detected in four peak fractions (*P-1–P-4*) in TOYOPEARL CM-650M chromatography performed for the proteins obtained by the ammonium sulfate fractionation (Figure 1). The N-terminal amino-acid sequences of major proteins in *P-1–P-4* fractions were analyzed with the samples blotted to PVDF membranes after SDS-PAGE. According to BLAST search analyses, the 40 kDa protein in *P-1* fraction (N-terminal sequence: RLHIQNGHFVLNGQRVFLSG) was identified as *A. kurodai* β-mannanase AkMan (Zahura et al., 2010). The 21 kDa protein in *P-2* fraction (N-terminal sequence: EQKCQPNSHGVRVYQGKKCA) was considered to be a GHF45 cellulase corresponding to 21K cellulase previously reported by Tsuji et al. (2013). The 45 kDa protein in *P-4* fraction (N-terminal sequence: AKNYGQALGLSIKFYEAQ) was regarded as a GHF9 cellulase similar to *H. discus hannai* HdEG66 (Suzuki et al., 2003) and 45K cellulase reported by Tsuji et al. (2013). While 38 kDa protein (N-terminal sequence: RLTVSGKTFRLNNQQVFLSG) was regarded as the β-mannanase-like protein that had been annotated in *A. california* genome (GenBank accession number, XP_005100017). The 21 kDa cellulase in *P-2* fraction was recovered in high yield, while the GHF9-type cellulase in *P-4* fraction poorly recovered. The GHF9-type cellulase exhibited similar properties as abalone cellulase HdEG66 (Suzuki et al., 2003) and Aplysia 66K cellulase (Tsuji et al., 2013). Therefore, in the present study, we focused on the 21 kDa cellulase in *P-2* fraction to characterize it as a GHF45 cellulase.

**Figure 2:**
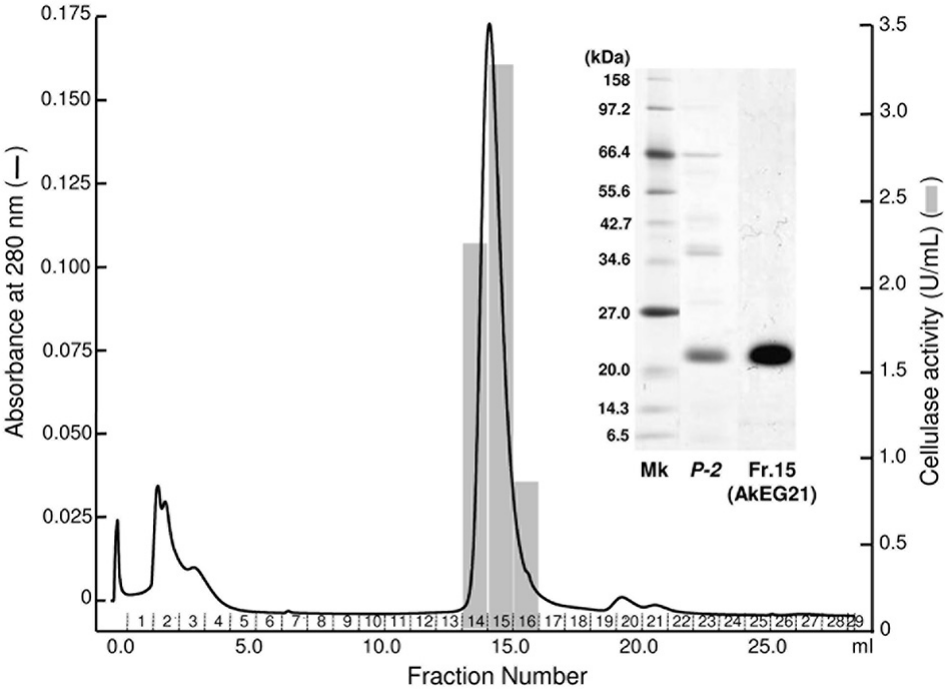
**Purification of AkEG21 by Superdex 200 gel filtration**. The *P-2* fraction obtained in the TOYOPEARL CM-650M column chromatography was subjected to gel filtration through Superdex 200 10/300 GL. Fractions 14–16 were pooled as the purified AkEG21. SDS-PAGE for cellulase preparations from *A. kurodai* is shown in the inset. Mk, marker protein; *P-2*, the sample after TOYOPEARL CM-650M chromatography; *Fr. 15*, purified AkEG21.

**Figure 3:**
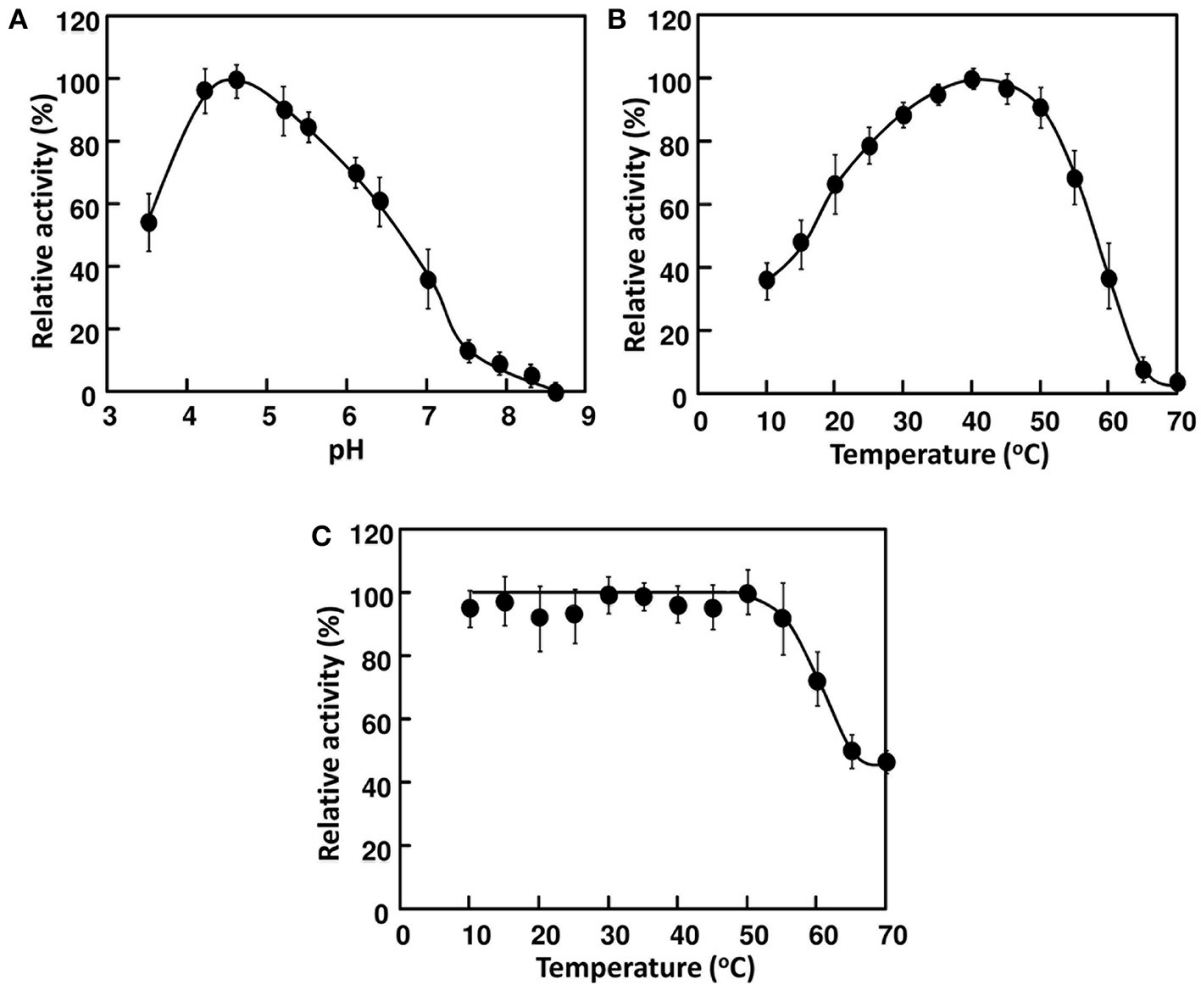
**Effects of pH and temperature on the activity of AkEG21. (A)** pH dependence of AkEG21 was examined at 30°C using reaction mixtures adjusted to pH 4–10 with 50 mM sodium phosphate buffer. **(B)** Temperature dependent activity of AkEG21 was measured at 10–60°C in a reaction mixture containing 0.5% CMC and 10 mM sodium phosphate (pH 6.0). **(C)** Thermal stability of AkEG21 was assessed by measuring the activity remaining after the heat-treatment at 10–70°C for 30 min.

The 21 kDa cellulase in *P-2* fraction was purified by gel-filtration through Superdex 200 (Figure 2). In this chromatography, the 21 kDa cellulase eluted as a single peak showing a single band on SDS-PAGE. Thus, we named this enzyme AkEG21 (*Aplysia kurodai* endo-β-1,4-glucanase with 21 kDa). By the above purification procedure, AkEG21 was purified at a yield of 3.3% with the specific activity 67.3 U/mg (Table 1). Optimal pH of AkEG21 was 4.5 and more than 80% of maximal activity was retained in a pH range from 4.3 to 5.6 (Figure 3A). AkEG21 showed an optimal temperature at around 40°C and it was resistant to the incubation at 55°C for 30 min. The temperature that caused a half inactivation of AkEG21 during 30 min incubation was ~65°C (Figures 3B,C). These results indicated that AkEG21 was relatively heat-stable among the molluscan cellulases reported so far.

**Table 1:** **Summary of purification of AkEG21**.

**Purification**	**Total protein (mg)**	**Total activity (U)**	**Specific activity (U^a^/mg)**	**Purification (fold)**	**Yield (%)**
Crude enzyme^b^	1440	11,552	8.0	1	100
AS^c^	798	8912	11.2	1.4	77.2
CM^d^	8.9	529.8	59.8	7.5	4.6
Gel filtration^e^	5.6	376.1	67.3	8.4	3.3

a *One unit (U) of cellulase was defined as the amount of enzyme that produces reducing sugar equivalent to 1 μmol of glucose per minute from 0.5% CMC*.

b *Crude enzyme after the dialysis against 2 mM sodium phosphate buffer (pH 7.0)*.

c *Fraction precipitated between 40 and 60% saturation of ammonium sulfate*.

d *Active fraction obtained by TOYOPEARL CM-650M chromatography*.

e *AkEG21 purified by the gel filtration through Superdex 200 10/300 GL*.

**Figure 4:**
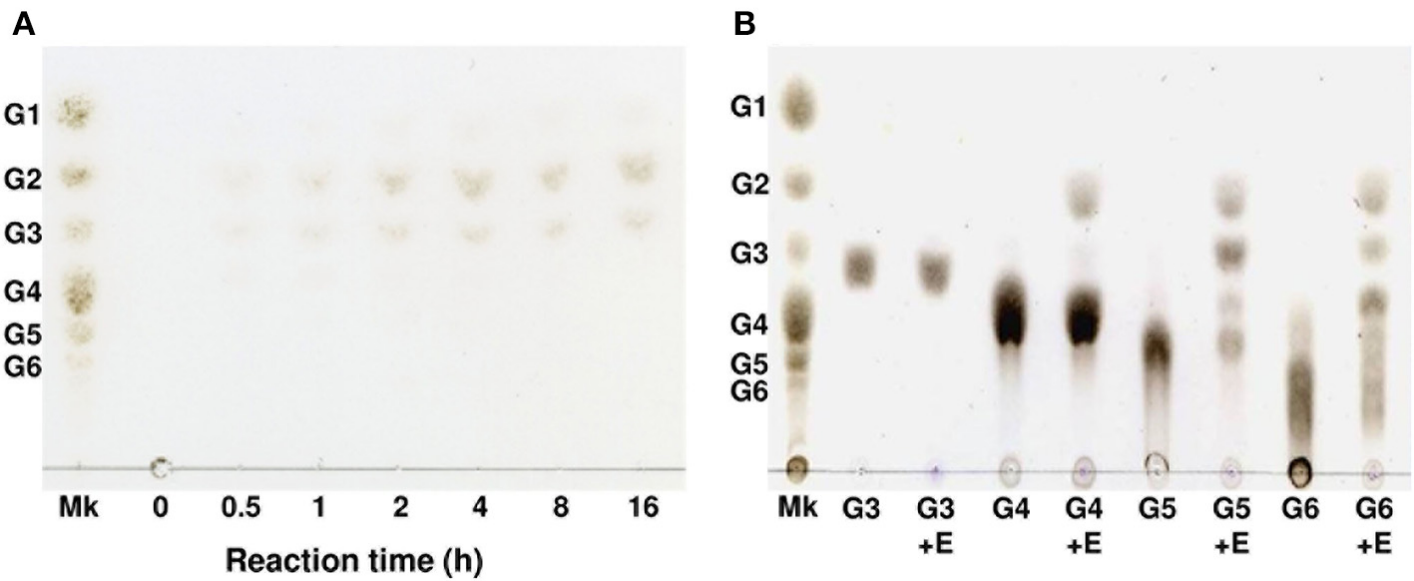
**Thin-layer chromatography of degradation products of phosphoric acid-swollen cellulose and cello-oligosaccharides produced by AkEG21. (A)** The reaction mixture of 1.0 mL containing 2.5 mg of phosphoric acid-swollen cellulose, 10 mM sodium phosphate (pH 6.0), and 7 U of AkEG21 was incubated at 37°C for up to 16 h. The supernatant of the reaction mixture was subjected to TLC. **(B)** The reaction mixture of 0.05 mL containing 0.05 mg of G3–G6, 10 mM sodium phosphate (pH6.0), and 7 U of AkEG21 was incubated at 30°C for 24 h, then subjected to TLC. G1, glucose; G2, cellobiose; G3, cellotriose; G4, cellotetraose; G5, cellopentaose; G6, cellohexaose. Mk, marker sugars.

Degradation products of cellulose and cellooligosaccharides produced by AkEG21 were analyzed by TLC. As shown in Figure 4A, AkEG21 degraded amorphous cellulose producing cellobiose and cellotriose. Cellooligosaccharides larger than cellotriose were not detected in the reaction products even after 16 h incubation. On the other hand, AkEG21 showed high activity toward hexaose (G6) and pentaose (G5) and weak activity toward tetraose (G4), but no activity toward triose (G3) (Figure 4B). AkEG21 readily degraded G5 to G2 and G3 (plus trace amount of G4 and glucose), and degraded G6 to G2 and G4 along with small amount of G3. These degradation profiles were practically unchanged even in the longer reaction time and higher enzyme concentrations although the amounts of the products were increased (data not shown). These results indicate that AkEG21 is an endo-β-1,4-D-glucanase (EC 3.2.1.4). However, readily production of cellobiose and small amount of cellotriose from amorphous cellulose without larger intermediate oligosaccharides may indicate that this enzyme can act as cellobiohydrolase-like enzyme as suggested by Tsuji et al. (2013).

### Cloning of AkEG21 cDNA

The N-terminal amino-acid sequence of AkEG21 was determined as EQKCQPNSHGVRVYQGKKCA- by the protein sequencer (Table 2). This sequence shared ~40, 40, 55, and 60% amino-acid identities to those of Eg from *M. edulis* (DDBJ accession number, CAC59694), CjCel45A from *C. japonica* (DDBJ accession number, AB468959), endo-β-1,4-glucanase 1 from *H. discus discus* (DDBJ accession number, EF103350) and EG27I from *A. crossean* (DDBJ accession number, EF471315), respectively. The sequences of tryptic and lysylendopeptidyl fragments of AkEG21 also showed 62–85% identities with the corresponding sequences of above molluscan cellulases (Table 2). Such high sequence similarities of partial amino-acid sequences between AkEG21 and other GHF45 cellulases suggested that AkEG21 also belongs to GHF45.

**Table 2:** **N-terminal and internal amino-acid sequences of AkEG21**.

**Peptides^a^**	**Amino-acid sequences**	**Similarity (%) to other molluscan GHF45 enzymes^b^**
AkEG21	EQKCQPNSHGVRVYQGKKCA-	*A. crossean* (60.0%, 17–36)
		*H. discus discus* (55.0%, 18–36)
L-1	VNDHGYEAHFDLQNNK	*A. crossean* (68.8%, 143–158)
		*H. discus discus* (68.8%, 141–156)
L-2	LTPTGGFVPGNGK	*A. crossean* (84.6%, 95–107)
		*H. discus discus* (61.5%, 92–104)
T-1	CQPNSHGVR	*A. crossean* (77.8%, 20–28)
		*H. discus discus* (44.4%, 21–28)
T-2	VWCGQSGKPGTNK	*A. crossean* (61.5%, 130–142)
		*H. discus discus* (46.2%, 128–140)
T-3	YNDGHR	*A. crossean* (66.7%, 41–46)
		*H. discus discus* (83.3%, 41–46)

a *L-1 and L-2, Lysylendopeptidyl fragments; T-1–T-3, tryptic fragments*.

b *Residue numbers for corresponding regions in the sequences of A. crossean and H. discus discus (see **Figure 6**) are also shown in the parentheses*.

**Figure 5:**
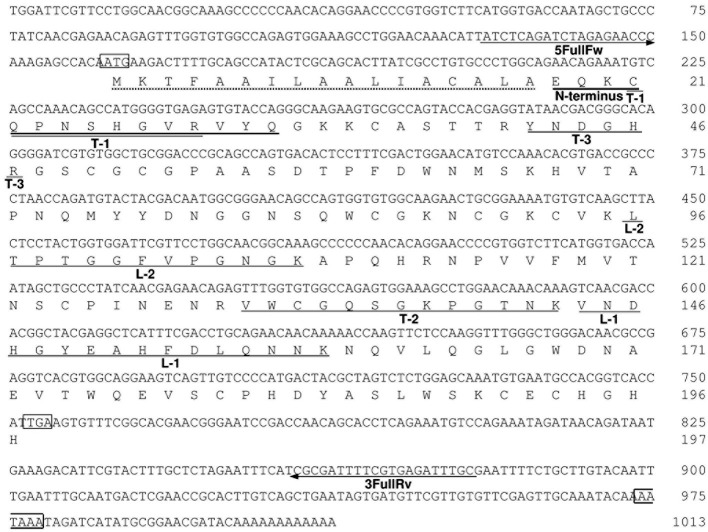
**The nucleotide and deduced amino-acid sequences of AkEG21**. Residue numbers for nucleotides and amino-acids are indicated in the right of each row. The translational initiation codon ATG, termination codon TGA, and a putative polyadenylation signal AATAAA are boxed. A putative signal peptide is indicated by a dotted underline. The amino-acid sequences determined with intact AkEG21 (N-terminus) and peptide fragments (L-1, L2, T1-T-3) are indicated with solid lines under the amino-acid sequence. The positions of 5FullFw and 3FullRv primers are indicated with arrows under the nucleotide sequence. The sequence data are available from the DNA Data Bank of Japan with an accession number, AB920344.

To determine the entire amino-acid sequence of AkEG21, we amplified AkEG21 cDNA by the PCR using degenerated forward and reverse primers, cDNA-1(Fw) and cDNA-1(Rv), respectively (Table 3). The amplified cDNA comprised 405 bp and encoded an amino-acid sequence of 135 residues. Then, 3RACE-cDNA of 519 bp covering the 3′-terminal region was amplified by 3′-RACE PCR with specific primers designed on the basis of the nucleotide sequence of the first amplified cDNA. Finally, 5RACE-cDNA of 363 bp covering 5′-terminal region was amplified by 5′-RACE PCR with a series of specific primers synthesized on the basis of the sequence of the amplified cDNA. By overlapping the nucleotide sequences of 5RACE-cDNA, first amplified cDNA and 3RACE-cDNA in this order, the nucleotide sequence of total 1013 bp including the complete translational region of AkEG21 was determined (Figure 5). The transcription-initiation codon (ATG) was found in the nucleotide positions from 162 to 164, while the termination codon (TGA) was in 753–755. Accordingly, the coding region of AkEG21 cDNA was found to locate in the nucleotide positions from 162 to 752 and encode 197 amino-acids. All the partial amino-acid sequences determined with peptide fragments, L-1, L-2, and T-1–T-3 (Table 2), were found in the deduced sequence (Figure 5). A putative polyadenylation-signal sequence (AATAAA) located at 22 nucleotides upstream from the poly (A)^+^ tail. This suggested that the origin of AkEG21cDNA was not intestinal prokaryotes but eukaryote, i.e., *Aplysia* itself. The N-terminus of mature AkEG21 protein was identified as Glu18 in the deduced sequence indicating that the N-terminal region of 16 residues except for initiation Met was the signal peptide for secretion. Indeed, KTFAAILAALIACALA located in the N-terminus of the deduced sequence was predicted as the signal peptide by SignalP 4.1 server (http://www.cbs.dtu.dk/services/SignalP/). Accordingly, the mature AkEG21 was concluded to comprise 180 amino-acid residues with the calculated molecular mass of 19,854.0 Da (Figure 5). The nucleotide and the deduced amino-acid sequences are available from the DNA Data Bank of Japan with the accession number AB920344.

**Table 3:** **Primers used for amplification of AkEG21-cDNA**.

**Primer names**	**Sequences**
**PCR**	
cDNA-1(Fw)	5′-AARACNCARCCNAAYWSNCAYGGNGTNMGNATG-3′^a^ (KTQPNCHGVRM)^b^
cDNA-1(Rv)	5′-TCRAARTGNGCYTCRTANCCRTGRTC-3′ (DHGYEAHFD)
**3′-RACE**	
3Fw	5′-TGACCAATAGCTGCCCTATC-3′
3Adapt	5′-CTGATCTAGAGGTACCGGATCC-3′
**5′-RACE**	
5RACE(Fw2)	5′-TGGATTCGTTCCTGGCAACG-3′
5RACE(Rv2)	5′-GTTTGGACATGTTCCAGTCG-3′
**CONFIRMATION**	
5FullFw	5′-ATCTCAGATCTAGAGAACCC-3′
3FullRv	5′-CGCAAATCTCACGAAAATCGCG-3′

a *R, adenine or guanine; Y, cytosine or thymine; W, adenine or thymine; S, cytosine or guanine; M, adenine or cytosine; N, adenine or guanine or cytosine or thymine*.

b *Amino-acid sequences used for designing the degenerated primers are in the parentheses*.

**Figure 6:**
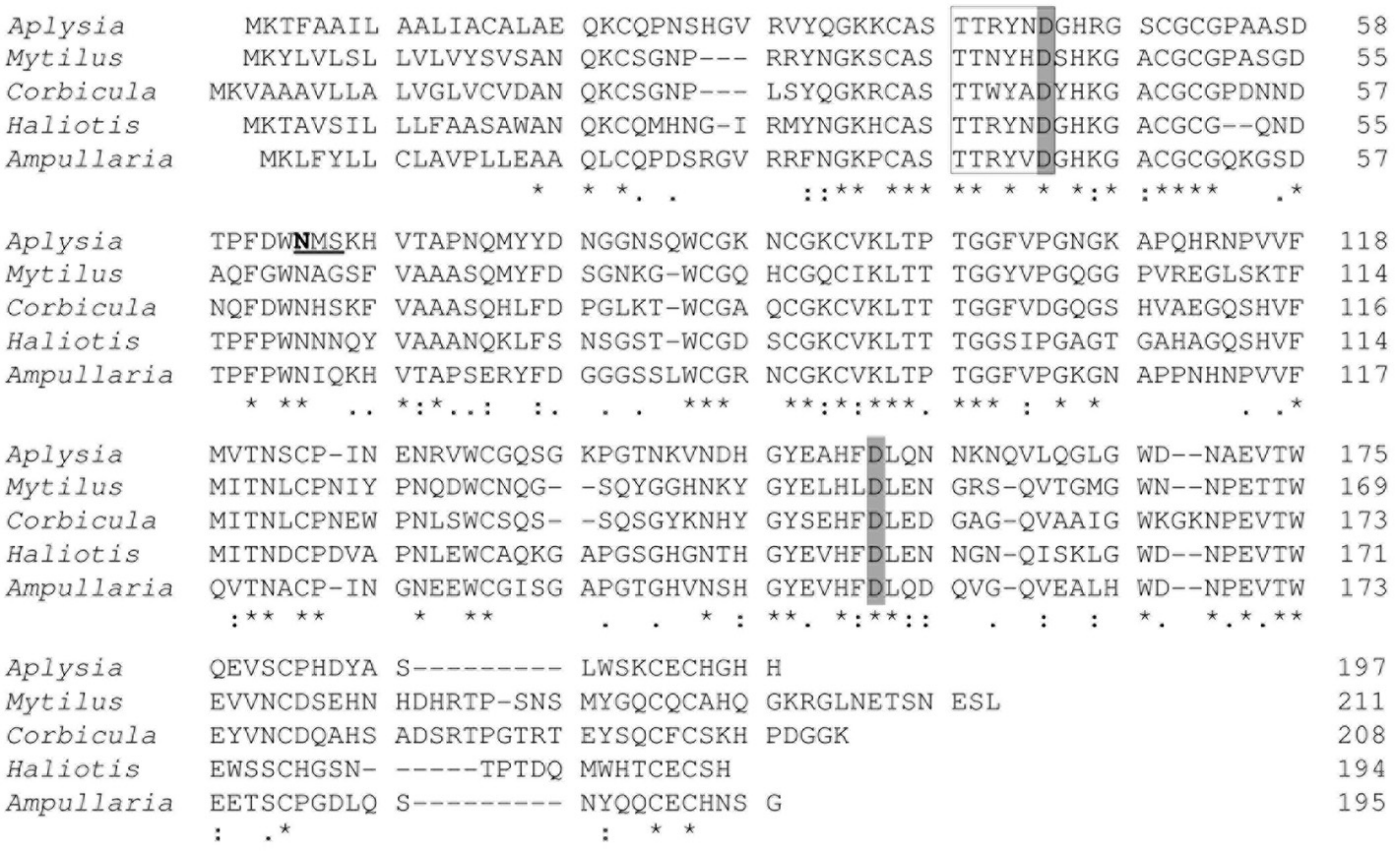
**Alignment of the amino-acid sequences of AkEG21 and other molluscan GHF45 cellulases**. The amino-acid sequence of AkEG21 (DDBJ accession number, AB920344) was aligned with those of endoglucanase (Eg) from *M. edulis* (DDBJ accession number, CAC59694), CjCel45A from *C. japonica* (DDBJ accession number, AB468959), endo-β-1,4-glucanase 1 from *H. discus discus* (DDBJ accession number, EF103350), and EG27I from *A. crossean* (DDBJ accession number, EF471315). Identical, highly conservative, and conservative residues among sequences are indicated by asterisk (^*^), colon (:), dot (.), respectively. The consensus amino-acid sequence and residues in catalytic sites of GHF45 cellulases are boxed and shaded, respectively. The putative N-glycosylation site is underlined and N-glycosylated residue is indicated with bold letter.

The amino-acid sequence of AkEG21 was aligned with those of other molluscan GHF45 cellulases (Figure 6) and 47, 49, 54, and 62% identities were calculated between AkEG21 and Eg from *M. edulis* (GenBank accession number, CAC59695), CjCel45A from *C. japonica* (GenBank accession number, BAH23793), endo-β-1,4-glucanase 1 from *H. discus discus* (GenBank accession number, ABO26608), and EG27I from *A. crossean* (GenBank accession number, ABR92637), respectively. The consensus amino-acid sequence T-T-R-Y-X-D that has been shown to take part in the catalytic site of GHF45 enzymes (Girard and Jouanin, 1999; Guo et al., 2008) was conserved in AkEG21 as Thr39–Asp44. The N-glycosylation site (Asn-X-Thr/Ser) was also conserved as Asn64–Ser66 where Ans64 was the N-glycosylation residue according to the analyses with NetNGlyc 1.0 server (http://www.cbs.dtu.dk/services/NetNGlyc/). Twelve Cys residues that form six disulfide bonds stabilizing the catalytic domain were also conserved in AkEG21. Two Asp residues that function as catalytic nucleophile and proton donor in GHF45 enzymes were conserved as Asp44 and Asp154, respectively, in AkEG21. These features in the amino-acid sequence of AkEG21 indicate that this enzyme belongs to GHF45.

### Phylogenetic analysis

**Figure 7:**
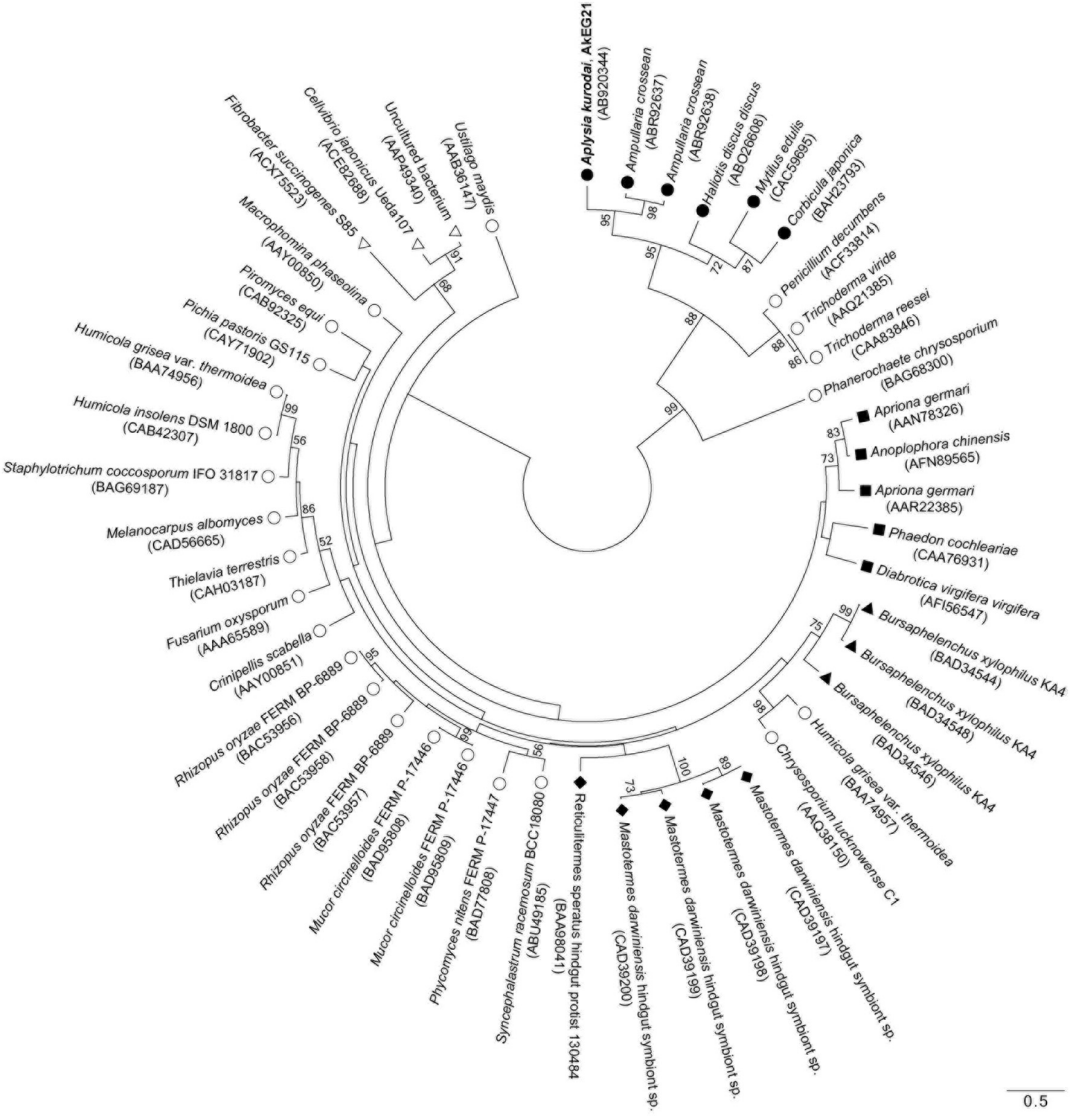
**Phylogenic relationship for GHF45 cellulases**. An unrooted phylogenetic tree for catalytic domains of AkEG21 and GHF45 enzymes from CAZy database was generated using MEGA 6 software. Bootstrap values over 50 are indicated on the branches. The scale bar indicates 0.50 amino-acid substitutions. Symbols shown with scientific names of organisms indicate as follows: solid circles, mollusks; open circles, fungi; solid squares, insects; solid triangles, nematode; solid diamonds, protists; open triangles, bacteria. GenBank accession numbers for each sequence data are shown in the parentheses after the names of organisms.

To reveal the structural relationship between AkEG21 and other GHF45 cellulases, phylogenetic analysis was performed using amino-acid sequence data of GHF45 cellulases from mollusk, fungi, insects, nematode, protists and bacteria. The tree topology drawn by the maximum likelihood analysis revealed that molluscan GHF45 cellulases are assembled as a large clade (bootstrap values above 50%) with some fungal enzymes (Figure 7). Whereas, enzymes from insects, nematode, protists, bacteria and some other fungi formed another paraphyletic group. These clustering results suggest that molluscan GHF45 cellulases have been deviated from other animal cellulases but closely related to some fungal cellulases.

## Discussion

The ocean that covers 70% of surface on the Earth is rich in biodiversity, e.g., organisms from 34 of 38 animal phyla are living in the ocean. Through the adaptation to diverse physical and chemical conditions of marine environments, marine organisms are believed to have deviated along with acquiring specific phenotypes. Thus, the marine organisms have become capable of producing variety of characteristic chemical compounds relating to lipids, proteins and carbohydrates, as a result of adaptation to marine environments. Such marine bio-products are promising materials for functional food additives, pharmaceutics, cosmetics, industrial materials, energy sources, etc. Among the marine bio-products, polysaccharides produced by marine algae, e.g., agar, carrageenan and alginate, which have already been used as gelling agents, viscosifiers and dietary fibers in food, are also important materials for producing functional oligosaccharides and fermentable sugars (Nishida et al., 2007; Kumagai and Ojima, 2009; Rahman et al., 2010; Zahura et al., 2010; Takeda et al., 2011; Wargacki et al., 2012; Tsuji et al., 2013; Yanagisawa et al., 2013; Enquist-Newman et al., 2014; Kumagai et al., 2014). Actually, enzymatically degraded seaweed polysaccharides were shown to exhibit beneficial activities to human (Deville et al., 2007; Wang et al., 2012; Thomas and Kim, 2013). Further, monosaccharides produced by the degradation of alginate were found to be available as a source material for ethanol fermentation (Takeda et al., 2011; Wargacki et al., 2012; Enquist-Newman et al., 2014). While, sea lettuce *Ulva pertusa* was also used as feedstock for acetone and ethanol (Yanagisawa et al., 2011; van der Wal et al., 2012). These trends have stimulated the exploration of new enzymes that convert seaweeds' polysaccharides to value-added materials.

Herbivorous mollusks produce various kinds of polysaccharide-degrading enzymes, e.g., alginate lyase (Wong et al., 2000; Shimizu et al., 2003; Suzuki et al., 2006; Rahman et al., 2010), mannanase (Zahura et al., 2010, 2011), laminarinase (Kozhemyako et al., 2004; Kovalchuk et al., 2006, 2009; Kumagai and Ojima, 2009, 2010; Pauchet et al., 2009), amylase (Kumagai et al., 2012) and cellulase (Suzuki et al., 2003; Guo et al., 2008; Sakamoto and Toyohara, 2009). Among these enzymes, cellulase appears to be most widely distributed in mollusks (Elyakova, 1972; Nishida et al., 2007; Sakamoto and Toyohara, 2009; Ravindran et al., 2010; Nagano et al., 2011). However, information about enzymatic properties and physiological significance of molluscan cellulases are still poorly understood compared with the enzymes from microbes (Tomme et al., 1995; Hong et al., 2002; Masuda et al., 2006; Fibriansah et al., 2007). In the present study, we isolated a GHF45-type cellulase AkEG21 from the common sea hare *A. kurodai* and determined its general properties.

AkEG21 showed an optimum pH at around 4.5 and more than 80% of maximal activity retained at pH range from 4.3 to 5.6. This pH range is consistent with the pH range of the digestive fluid of *A. kurodai*, i.e., pH 4–6 (Zahura et al., 2011; Tsuji et al., 2013). Although optimal pH of microbial cellulases is known to vary from acidic to alkaline range (Hurst et al., 1977; Ito et al., 1989; Park et al., 2002), those of animal GHF45 cellulases are usually in acidic pH range. For example, optimal pHs of EG27 from *A. crossean* (Li et al., 2005) and Cel45A from *M. edulis* (Xu et al., 2000) were shown at around 4.4–4.8. While, heat stability of AkEG21 was found to be considerably high, i.e., it retained more than 80% of maximal activity after the pre-incubation at 55°C for 30 min and was not completely inactivated even at 70°C (Figure 3). Such high heat stability was also reported in EG27 from *A. crossean* (Li et al., 2005), Cel45A from *M. edulis* (Xu et al., 2000) and 21K cellulase from *A. kurodai* (Tsuji et al., 2013). EG27 retained ~85% of maximal activity after the incubation at 60°C for 24 h (Li et al., 2005) and Cel45A retained more than 70% of the activity after the incubation in boiling water bath for 10 min (Xu et al., 2000). Such stabilities of GHF45 cellulases in acidic and high temperature conditions may be due to the formation of plural disulfide bonds in the catalytic domain. Such stability of GHF45 cellulase will be advantageous in performing both basic researches and biotechnological applications. On the other hand, *M. edulis* cellulase Cel45A was reported to show an unusual psychrophilic feature, i.e., it retains 55–60% of its maximum activity even at 0°C (Xu et al., 2000). AkEG21 also showed relatively high activity in low temperature conditions, e.g., it retained ~40% of the maximal activity at 10°C (Figure 3). In this respect, molluscan GHF45 cellulases may be applicable for cellulose degradation in acidic and broad temperature conditions.

AkEG21 produced cellotriose and cellobiose as major products from amorphous cellulose and efficiently hydrolyzed cellohexaose and cellopentaose, and moderately cellotetraose, but not cellotriose and cellobiose. These indicated that AkEG21 recognized cellotetraose unit in cellulose chain and split the central glycosyl linkage of tetraose. Such substrate-recognition profiles of AkEG21 were essentially the same as those of from Cel45A from *M. edulis* (Xu et al., 2000) and 21K cellulase from *Aplysia* (Tsuji et al., 2013).

By the cDNA method, an entire amino-acid sequence of AkEG21 comprising 197 residues was predicted. The sequence of catalytic domain comprising 180 residues shared 47–62% amino-acid identities with the other molluscan GHF45 cellulases and conserved T-T-R-Y-X-D motif and two Asp residues which were identified as catalytic site and residues of GHF45 enzymes (Girard and Jouanin, 1999; Bourne and Henrissat, 2001; Guo et al., 2008) (Figure 6). AkEG21 possessed a typical N-glycosylation motif (Asn-X-Thr/Ser) at amino-acid positions of 64–66, and the Ans64 was predicted to be the N-glycosylation residue (Figure 6). The 21K cellulase from *Aplysia* was shown to be glycosylated (Tsuji et al., 2013). This indicated that AkEG21 was a glycosylated enzyme. Cel45A from *M. edulis* (Xu et al., 2000) and CjCel45A from *C. japonica* (Sakamoto and Toyohara, 2009) were also suggested to be glysocylated at the N-glycosylation sites, while no N-glycosylation site was found in GHF45 cellulase from *A. crossean* (Guo et al., 2008) and *H. discus discus* (GenBank accession number, ABO26608) (Figure 6). On the other hand, coleopteran GHF45 cellulases, e.g., Ag-EGase I (contain 2 N-glycosylation sites) and Ag-EGase II (contain 3 N-glycosylation sites) from *Apriona germari*, and Oa-EGase II (contain 2 N-glycosylation sites) from *Oncideresalbomarginata chamela* were found to be N-glycosylated and the N-glycosylations were important for secretion and enzyme activity (Wei et al., 2006; Calderon-Cortes et al., 2010). Previous report showed that 90% of proteins possessing the sequence Asn-X-Ser/Thr were glycosylated (Gavel and von Heijne, 1990). The roles of glycosylation are known to vary from protein to protein (Bisaria and Mishra, 1989; Wang and Gao, 2000). It is necessary to examine the physiological significance of the N-glycosylation in AkEG21 using recombinant enzymes expressed in prokaryote cells where no glycosylation takes place.

AkEG21 contained 12 Cys residues. This suggested that occurrence of six disulfide bonds in AkEG21, which may structurally stabilize the catalytic domain. All the molluscan GHF45 possess 12 Cys residues in common positions, suggesting that the stabilization by 6 disulfide bridges is a common feature among the GHF45 cellulases. Extremely high thermal stability of Cel45A from *M. edulis*, which withstands the heat-treatment at 100°C for 10 min (Xu et al., 2000), may be derived from such disulfide bonds. AkEG21 was also considerably heat stable probably due to the multiple disulfide formations.

In most organisms, cellulases are produced as modular enzymes made up of a catalytic domain and cellulose-binding domain(s) (CBD) that facilitates adsorption of the catalytic domain to insoluble cellulose (Gilkes et al., 1991; Henrissat and Davies, 2000). However, AkEG21 lacked cellulose-binding domain (CBD). Lack of CBD was also the cases of Cel45A from *M. edulis* (Xu et al., 2000), EG27 from *A. crossean* (Guo et al., 2008) and CjCel45 from *C. japonica* (Sakamoto and Toyohara, 2009). Physiological meaning of the lack of CBD in molluscan GHF45 cellulases is currently obscure; however, low affinity of enzyme to cellulose substrate may rather suitable for the turnover of enzyme in the digestive fluid to digest amorphous seaweed cellulose.

Kinds of animal digestive enzymes appeared to be closely related to the staple foods of animals (Baldwin, 1949). However, distribution of cellulase in animal kingdom was found to be more closely related to their phylogenetic relationships than their feeding habits (Yokoe and Yasumasu, 1964). GHF45 cellulases have been found in fungi, bacteria, protists, and some invertebrate animals (Henrissat and Bairoch, 1993; http://www.cazy.org/Glycoside-Hydrolases.html). Phylogenetic analysis revealed that molluscan GHF45 cellulases and some fungal enzymes were clustered as a distinct group (Figure 7). Such clustering of molluscan GHF45 cellulases suggested that they have evolved from the same origin. Relatively close relation between molluscan cellulases and fungal cellulases suggests that molluscan enzymes were acquired by horizontal gene transfer from fungi as suggested by Sakamoto and Toyohara (2009). On the other hand, presence of potential N-glycosylation sites in all molluscan GHF45 cellulases is in common with some coleopteran cellulases may suggest that the molluscan cellulases share the common ancestor with insect GHF45 cellulases and have diverged from them during the evolutionary process (Davison and Blaxter, 2005; Watanabe and Tokuda, 2010). Rigorous investigation is necessary before concluding that the animal cellulases are acquired by horizontal gene transfer from fungi (Ochman et al., 2000; Genereux and Logsdon, 2003).

Besides AkEG21, a GHF9 cellulase of 45 kDa was also found in the digestive fluid of *A. kurodai* (see Figure 1). Occurrence of multiple cellulase genes belonging to different GHFs in mollusks has already been reported (Zhang et al., 1999; Wang et al., 2003; Li et al., 2005; Sakamoto et al., 2007; Guo et al., 2008; Sakamoto and Toyohara, 2009) and synergistic action of multiple cellulases was recently reported (Tsuji et al., 2013). It may be reasonable to consider that herbivorous mollusks rely on plural cellulases to degrade cellulose to obtain carbohydrate nutrient from seaweeds. Indeed, the GHF9 cellulase of *A. kurodai* exhibited relatively higher specific activity compared with the GHF45 cellulase AkEG21 upon degradation of amorphous cellulose (Tsuji et al., 2013). Such differences in enzymatic properties were attributed to the differences in enzymatic parameters (Tsuji et al., 2013). The protein-engineering study on AkEG21 for application of this enzyme as biocatalyst for degradation of cellulosic materials from seaweeds is now under the investigation.

### Conflict of interest statement

The authors declare that the research was conducted in the absence of any commercial or financial relationships that could be construed as a potential conflict of interest.
